# A connectivity-based parcellation improved functional representation of the human cerebellum

**DOI:** 10.1038/s41598-019-45670-6

**Published:** 2019-06-24

**Authors:** Yudan Ren, Lei Guo, Christine Cong Guo

**Affiliations:** 1 School of Automation, Northwestern Polytechnical University, Xi’an, China; 2 QIMR Berghofer Medical Research Institute, Brisbane, Australia; 3 School of Information Science and Technology, Northwest University, Xi’an, China

**Keywords:** Computational neuroscience, Cognitive neuroscience

## Abstract

The cerebellum is traditionally well known for its role in motor learning and coordination. Recently, it is recognized that the function of the cerebellum is highly diverse and extends to non-motor domains, such as working memory, emotion and language. The diversity of the cerebellum can be appreciated by examining its extensive connectivity to the cerebral regions selective for both motor and cognitive functions. Importantly, the pattern of cerebro-cerebellar connectivity is specific and distinct to different cerebellar subregions. Therefore, to understand the cerebellum and the various functions it involves, it is essential to identify and differentiate its subdivisions. However, most studies are still referring the cerebellum as one brain structure or by its gross anatomical subdivisions, which does not necessarily reflect the functional mapping of the cerebellum. We here employed a data-driven method to generate a functional connectivity-based parcellation of the cerebellum. Our results demonstrated that functional connectivity-based atlas is superior to existing atlases in regards to cluster homogeneity, accuracy of functional connectivity representation and individual identification. Furthermore, our functional atlas improves statistical results of task fMRI analyses, as compared to the standard voxel-based approach and existing atlases. Our detailed functional parcellation provides a valuable tool for elucidating the functional diversity and connectivity of the cerebellum as well as its network relationships with the whole brain.

## Introduction

Generating an accurate map of the human brain has been a central focus for neuroscientists. Much efforts have been made to map the cerebral cortex, which uncovered a great degree of functional complexity and diversity^1^. The cerebellum, on the other hand, has been traditionally thought to be a uniform structure that primarily supports motor function^2,3^. A growing number of neuroimaging and clinical neuroscience studies, however, have convincingly demonstrated that the cerebellum comprises functional subdivisions that contribute to a large diversity of functions beyond the motor domain^4,5^, such as language processing^6–8^, working memory^8–10^, executive function^11,12^ and emotion processing^13,14^. Recent naturalistic fMRI research also demonstrated that the cerebellum is involved with dynamic perceptual and affective processes during film viewing^15^. The diverse functions of the cerebellum are further evident by its reciprocal connections with the cerebrum, which not only encompasses the motor cortex but also parietal and prefrontal cortices that support high-order cognitive processes^6,11,16,17^. Therefore, the function of the human cerebellum is likely to present a similar level of diversity and complexity as the cerebral cortex. To further elucidate this diverse function and connectivity, a detailed functional parcellation of the cerebellum is crucial.

Current atlases of the cerebellum are most based on its gross anatomy^18,19^. The cerebellum has distinctive anatomical subdivisions - the cerebellar lobules as defined by the cerebellar fissures^20^. Recent efforts to refine the cerebellar atlas mostly focused on automated methods to normalize and register the morphology of the cerebellum and thus identify the cerebellar lobules^18,19^. However, morphological or structural boundary might not necessarily define functional specialization^16,18,19^. In fact, not much evidence suggests that each cerebellar lobule acts as a functionally homogenous entity: most fMRI studies in the cerebellum reported functional activations at a subset of a lobule or across two lobules^15,21^. Furthermore, there is little overlap between the cerebellar lobules and the functional networks within the cerebellum based on resting state connectivity^16^. Therefore, structural atlases might fall short for examining the function and connectivity of the cerebellum. To move the field forward, we need to develop an atlas that represents functional parcellation of the cerebellum.

While several existing whole-brain functional parcellations encompass the cerebellum, the cerebellar parcels are relatively coarse and not necessarily optimized by the parcellation algorithms. To bridge this gap, we here generated a functional parcellation of the cerebellum based on resting state functional connectivity. Applying a data-driven method to the Human Connectome Project (HCP) resting state dataset, we parcellated the cerebellum into functionally homogeneous clusters. We also systematically compared this functional parcellation with the widely used anatomical atlas – the Spatially Unbiased Atlas Template, and two cerebellar parcellations based on resting state functional connectivity^16,22,23^ - in terms of ROI homogeneity, accuracy of functional connectivity representation and individual identification. Finally, we tested whether this parcellation could improve statistical analyses of task fMRI dataset over voxel-based approaches, and further compared its performance of task fMRI studies with existing atlases.

## Materials and Methods

### Overview

**Figure 1:**
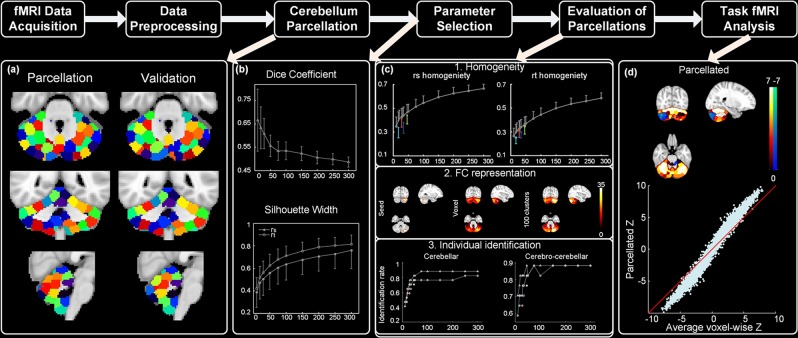
The overview of our pipeline. The atlases illustrated in (**a**) contain 100 clusters.

The overview of our analytical pipeline is illustrated in Fig. 1. First, we assigned two independent datasets as ‘parcellation’ and ‘validation’ group, and applied the parcellation method to these two groups separately (Fig. 1a). To determine the appropriate number of clusters for the parcellation, we repeated the parcellation with a series of cluster numbers and evaluated how cluster number affects the reproducibility (Dice coefficient) and homogeneity (silhouette width) of the parcellation results (Fig. 1b). Furthermore, we compared our functional atlases against previously established cerebellar atlases, namely the Spatially Unbiased Atlas Template, Shen’s functional parcellation and Buckner’s 17 networks, in regards to cluster homogeneity, accuracy of functional connectivity representation and individual identification (Fig. 1c). Finally, we assessed whether our atlas can improve statistical analysis of task fMRI data over voxel-based approach (Fig. 1d).

### Data acquisition and preprocessing

Minimally preprocessed resting state fMRI datasets for 57 healthy subjects (age: 26–35, 34 females) are obtained from the Q3 Data Release of Human Connectome Project (HCP)^24^, and consist of 1,200 frames of multiband, gradient-echo planar images (TA = 14 min and 33 s; TR = 720 ms; TE = 33.1 ms; flip angle = 52°; field of view = 208 × 180 mm; matrix = 104 × 90; 72 slices; voxel dimensions = 2 mm isotropic). Four resting state fMRI runs are acquired from each subject (left-right encoded and right-left encoded, two sessions of each), where all the runs in the first session are used as ‘parcellation’ group, and the ones in the second session served as ‘evaluation’ group. An independent un-preprocessed task fMRI datasets of seven tasks for 50 subjects (age: 22–35, 35 females) are obtained from the Q1 Data Release with the same acquisition parameters as resting state fMRI data other than the TA, including working memory, gambling, motor, language, social cognitive, relational processing and emotion processing tasks.

The HCP minimal preprocessing pipeline of resting state fMRI data includes artifact removal, motion correction and registration to standard space. We then applied additional preprocessing steps to the resting state fMRI data, including band-pass filtering, which is important to remove frequencies not implicated in resting-state functional connectivity^25,26^, and regression nuisance signals from the WM and CSF, which reflect non-neural fluctuations^27,28^. Resting state fMRI data were then smoothed with 2 mm full width half maximum Gaussian kernel, band pass filtered (0.0085–0.15 HZ), and further regressed out nuisance covariates including WM, CSF and motion parameters using the Data Preprocessing Assistant for Resting-state fMRI software (DPARSF)^29^. For the task fMRI datasets, our preprocessing pipeline included slice timing correction, motion correction, co-registration, normalization, spatial smoothing with 2 mm full width half maximum Gaussian kernel, and global drift removal (high-pass filtering), implemented by FSL FEAT^30^. Full details of data acquisition and the minimal preprocessing pipeline can be found in previous publications from HCP^31–34^.

An independent dataset is served as ‘validation’ group to further validate and evaluate our cerebellar atlases^35^. The dataset consists of two sessions with 3 months interval acquired from 17 healthy controls. In each session, subjects underwent 8-min resting state scan with eye closed on a whole-body 3T Siemens Trio MRI scanner. The details of scan parameters and preprocessing steps can be referred to our previous study^15,35^. All the fMRI datasets have been manually inspected to ensure the full coverage of the cerebellum.

### Functional parcellation of the cerebellum

A two-step data-driven clustering method is adopted to generate cerebellar atlases, where the first step generates subject specific similarity matrices and the second step finalizes the clustering at the group level^36–38^. In this study, we first applied this clustering method to the ‘parcellation’ group, generating cerebellar atlases with 10, 20, 30, 50, 75, 100, 150, 200, 250, 300 clusters for further parameter selection and evaluation. To further validate these results, we then generated atlases with a subset of cluster numbers (10, 100 and 300) for the ‘validation’ group to compare with ‘parcellation’ group.

To define subject-specific similarity matrix *W*, the similarity between the functional connectivity of each pair of voxels *v*_*i*_ and *v*_*j*_ within cerebellum is calculated as element *w*_*ij*_ of *W* (Eq. 1).

1 $${w}_{ij}=\{\begin{array}{cc}s({v}_{i},{v}_{j}) & {d}_{ij}\le \varepsilon \\ 0 & {d}_{ij} > \varepsilon \end{array}$$

The *d*_*ij*_ represents the distance between voxel *v*_*i*_ and *v*_*j*_, and the radius ε here is chosen to include the 26 nearest neighbors of a voxel. This three-dimension spatial constraint ensures the resulting clusters in atlas contain contiguous, spatially coherent voxels rather than spatially distributed voxels^39,40^. The similarity between voxels, *s*(*v*_*i*_, *v*_*j*_), is measured by Pearson’s correlation coefficient between time series of voxel *v*_*i*_ and *v*_*j*_ (r_t_)^38^. A threshold of 0.5 is applied to exclude negative and weak correlations. This r_t_ metric measures the temporal homogeneity within a cluster.

Group level clustering is accomplished by averaging the subject-specific similarity matrices, and the resulting matrix is clustered using the normalized cut spectral clustering (NCUT) algorithm (called group mean clustering method)^36–38^. An adjacency matrix A is constructed from the group clustering results, where the elements a_ij_ of A equals one if both voxel *v*_*i*_ and voxel *v*_*j*_ are contained in the same cluster and zero otherwise^38^. The NCUT algorithm shows superiority to other algorithms in terms of robustness to outliers^41,42^, favorable feasibility^43^, ability to incorporate constrains^39,40^. The details of NCUT algorithm have been described in previous studies^37,38^. The parcellation algorithm can be implemented by a publicly available toolbox pyClusterROI (http://ccraddock.github.io/cluster_roi/)^37^.

### Parameter selection

The clustering method requires the number of clusters to be pre-specified. Therefore, to determine the optimal number of clusters, we generated cerebellar atlases using a serial of cluster numbers and compared them in terms of reproducibility and cluster homogeneity. Two commonly used strategies are employed for this comparison: leave-one-out cross-validation (LOOCV) and Silhouette width^44,45^.

The LOOCV procedure selects one subject for testing and all remaining subjects for training. The procedure iterates till every subject is selected once and only once. In each iteration, clustering is performed on the testing subject (*m*^*th*^) and the training subjects separately. Then Dice’s coefficient was used to measure the similarity between the adjacency matrix generated from this *m*^*th*^ testing subject (A_m_) and the one generated from the training group (A_−m_, Eq. 2)^46^. Dice’s coefficient can range from zero to one, where zero corresponds to no similarity and one represents maximum similarity^46^. Dice’s coefficients derived from all iterations were then averaged to derive the reproducibility score of one atlas.

2 $${\rm{dice}}=\frac{2\cdot |{A}_{-m}\cap {A}_{m}|}{|{A}_{-m}|+|{A}_{m}|}$$

Silhouette width is used to quantify the functional homogeneity of clusters. The average similarity, a_k_, between every pair of voxels contained in the cluster *c*_*k*_ of atlas *C* ($C={\cup }_{k=1}^{{\rm{K}}}{c}_{k}$), is defined as Eq. 3:

3 $${a}_{k}=\frac{1}{{n}_{k}({n}_{k}-1)}\sum _{i,j\in {C}_{k},\,i\ne j}s({v}_{i},{v}_{j})$$

where *n*_*k*_ refers to the number of voxels assigned to cluster *c*_*k*_ and *s*(*v*_*i*_, *v*_*j*_) corresponds to the measurement of similarity between i^th^ voxel and j^th^ voxel — either the temporal similarity metric (r_t_) used for our parcellation, or a spatial similarity metric (r_s_) measured by Pearson’s correlation coefficient between functional connectivity maps generated by seed voxel i^th^ and j^th^ ^47^. Then, the average similarity between in-cluster and out-of-cluster voxels, b_k_, is

4 $${b}_{k}=\frac{1}{{n}_{k}(N-{n}_{k})}\sum _{i,\in {C}_{k}},\sum _{j\notin {C}_{k}}\,s({v}_{i},{v}_{j})$$

where N is the total number of voxels. The Silhouette width for atlas *C* is then defined as Eq. 5:

5 $$si(C)=\frac{1}{K}\sum _{k=1}^{K}\,\frac{{a}_{k}-{b}_{k}}{{\rm{\max }}\,\{{a}_{k},{b}_{k}\}}$$

Silhouette width can range from −∞ to ∞, where negative values represent improper parcellation and values near one or higher represent good parcellation. For each atlas, we calculated Silhouette width and Dice’s coefficient for each subject in the ‘parcellation’ group, and then averaged them across all subjects.

### Comparison to existing cerebellar atlases

We then compared the performance of our atlas against existing atlases or parcellation of the cerebellum, namely, a widely used anatomical atlas - the Spatially Unbiased Atlas Template, a whole-brain functional connectivity-based parcellation including cerebellum coverage - Shen’s functional parcellation and a gross cerebellar parcellation based on the cerebro-cerebellar connectivity - the Buckner’s 17 networks. Because the Buckner’s 17 networks atlas is coarse and does not separate the two hemispheres, we generated a Buckner’s 34 networks atlas by splitting each region at x = 0 into one on the left hemisphere and one on the right. The atlases are compared in three aspects: cluster homogeneity, accuracy of functional connectivity representation and individual identification. To avoid bias of evaluating the parcellation on the same dataset from which it was derived, the comparisons on cluster homogeneity, accuracy of functional connectivity representation and individual identification analyses are performed on the ‘validation’ group, and further on ‘evaluation’ group. As the ‘evaluation’ group generates consistent results as ‘validation’ group (Supplementary Figs 1–3, Supplementary Table 1), we here only show the comparison results from ‘validation’ group in the main text. In addition, we controlled for the influence of cluster numbers by generating additional atlases using our method with the same cluster numbers as these four atlases. The differences between our functional atlas and existing atlas were assessed by paired t-tests on each evaluation metric across all subjects. Bonferroni correction was applied in cases of multiple comparisons.

#### Cluster homogeneity

Cluster homogeneity is measured as the average r_t_^48^ and r_s_^47^ between every pair of voxels within a cluster. While r_t_ indicates temporal homogeneity within a cluster, r_s_ reveals spatial homogeneity of functional connectivity maps. For each atlas and each subject, we calculated average r_t_ and r_s_ across voxels within each cluster, and then averaged these values across clusters. For each atlas, the values were then averaged across all subjects.

#### Accuracy of functional connectivity representation

We first identified three seeds within the cerebellum by applying temporal concatenation group ICA to the resting state fMRI data in ‘parcellation’ group, as implemented by GIFT Matlab software^49^, and then used these seeds to calculate functional connectivity maps in the ‘validation’ group. This approach avoids potential bias in calculating functional connectivity maps on the same dataset from which the seeds are derived. The seed time series is derived by calculating the average BOLD time series across all voxels within the seed.

We then examined how well atlases-based functional connectivity maps match with voxel-wise functional connectivity maps, which have the highest spatial resolution and are regarded as ground truth. The voxel-wise functional connectivity maps are derived by correlating the seed time series with the time series of every voxel within cerebellum, as described previously^50^. The atlas-based functional connectivity maps are derived by correlating the seed time series with the time series of each cluster in the atlas – the average BOLD time series across all voxels within the cluster. The correlation coefficients of each cluster are then assigned to all voxels within the cluster so that the atlas-based functional connectivity maps have the same data dimension as the voxel-wise maps. The similarity between the atlas-based and the voxel-wise functional connectivity maps is measured by Pearson’s correlation coefficient and then averaged across subjects for comparison.

#### Individual identification

Here, we employed a functional connectivity-based identification method to assess the accuracy of individual identification using the cerebellar atlases^22^. Functional connectivity matrices were derived either between pairs of clusters within the cerebellum (cerebellar functional connectivity), or between cerebellar and cerebral clusters (cerebro-cerebellar functional connectivity) – cerebral clusters are the 200 ROIs based on the Craddock 2012 parcellation^37^.

For each cerebellar atlas, functional connectivity matrices were derived from the first session and second session of ‘validation’ group, respectively. Individual identification is then conducted between the two sessions, where one is used as the ‘target’ group and the other as the ‘database’ group. In each iteration, one subject’s functional connectivity matrix (either cerebellar or cerebro-cerebellar functional connectivity) from the target set is selected and compared against each of functional connectivity matrices in the database set. The similarity between each pair of matrices is defined as the Pearson correlation coefficient between their functional connectivity values. The matrix with maximum similarity in the database set is then identified as the predicted identity. If this predicted identity matches the true identity, the iteration is assigned a score of 1, and 0 if it does not. Each subject in target set is compared against the database set once and only once. The identification accuracy is defined as the percentage of iterations where the identity is correctly predicted out of the total number of iterations. For each atlas, we tested the identification using either the cerebellar or cerebro-cerebellar functional connectivity separately.

### Task fMRI analysis

We then tested the performance of our atlas in detecting functional activations during task fMRI^1^. To save computation, we only selected our 100-cluster atlas for this analysis. Each of seven task fMRI signals are first extracted and averaged for each cluster to derive cluster-level task fMRI data. Then, we conducted General Linear Model (GLM) analyses on both voxel-level and cluster-level task fMRI data using FSL toolbox^30^. FSL’s FILM algorithm is used to compute first level task fMRI statistics, and then FSL’s FLAME algorithm (FLAME1) is adopted to compute group level task fMRI z statistics. The HCP task fMRI z statistics maps for 86 contrasts are then derived from cluster-level and voxel-level data. For each voxel-level z statistics map, z values of all the voxels within each cluster are averaged to generate average voxel-wise z statistics. Finally, to verify if our atlas can improve signal-to-noise ratio (SNR) and statistical power in functional activation analyses, we compared the average voxel-wise z statistics against the parcellated z statistics (100 clusters × 86 task z statistics).

Furthermore, we then compared the performance of our atlas in detecting functional activations during task fMRI against existing cerebellar atlases, including the Spatially Unbiased Atlas Template (28 parcels), Shen’s functional parcellation (46 parcels), Buckner’s 17 networks (17 parcels) and Buckner’s 34 networks (34 parcels) atlas. The same procedure is applied to cluster-level task fMRI data to derive z statistics maps for 86 contrasts for all cerebellar atlases. We then examined how well atlases-based z statistics maps match with voxel-wise maps, which have the highest spatial resolution and are regarded as ground truth. Specifically, for each contrast and each atlas, the z values of each cluster are assigned to all voxels within the cluster so that the atlas-based z statistics maps have the same data dimension as the voxel-wise maps. The similarity between the atlas-based and the voxel-wise z statistic maps is measured by Pearson’s correlation coefficient. Finally, the differences between our functional atlas and existing atlas were assessed by paired t-tests on the similarity between atlas-based and voxel-wise z statistics maps across all the 86 contrasts.

### Ethical approval

All procedures performed in studies involving human participants were in accordance with the ethical standards stated in the 1964 Helsinki declaration and approved by the Washington University institutional review board.

### Informed consent

Written informed consent was obtained from all individual participants included in the study.

## Results

### Functional atlases of the cerebellum

We first parcellated the whole cerebellum into functionally and spatially coherent regions, using the ‘parcellation’ and ‘validation’ groups separately. For further parameter selection, we derived atlases with different cluster numbers.

**Figure 2:**
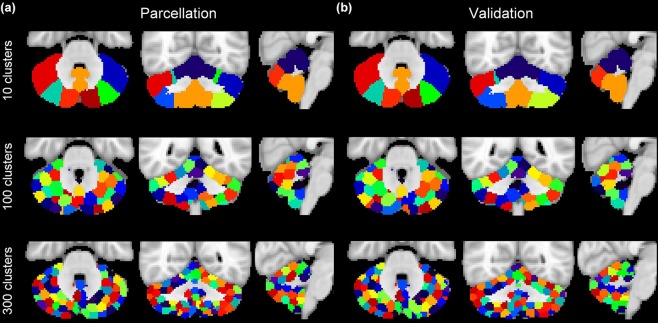
Cerebellar atlases for (**a**) ‘parcellation’ and (**b**) ‘validation’ groups containing 10, 100 and 300 clusters. Color codes are matched between the two groups as much as possible.

The parcellations based on functional connectivity are robust and consistent across the two groups, sharing similar shape, size and location (Fig. 2). In addition, the resultant ROIs show striking hemispheric symmetry, where most of lobules have qualitatively very similar clusters in both hemispheres. The majority of clusters in our atlases cover a subregion of a lobule or across lobules, consistent with a previous parcellation based on cerebro-cerebellar functional connectivity - Buckner’s 17 network^16^.

Clusters in the 10-cluster atlas are large and coarse, where voxels with distinct functions might be clustered into the same clusters (Fig. 2 upper panel). On the other hand, clusters in the 300-cluster atlas are small and scattered (Fig. 2 lower panel). The 100 cluster atlas appears to be a good compromise between these two extremes, whose clusters are of moderate shape and qualitatively symmetric between the two hemispheres (Fig. 2 middle panel). We then performed systematic and quantitative comparisons to select the optimal cluster number.

### Parameter selection

**Figure 3:**
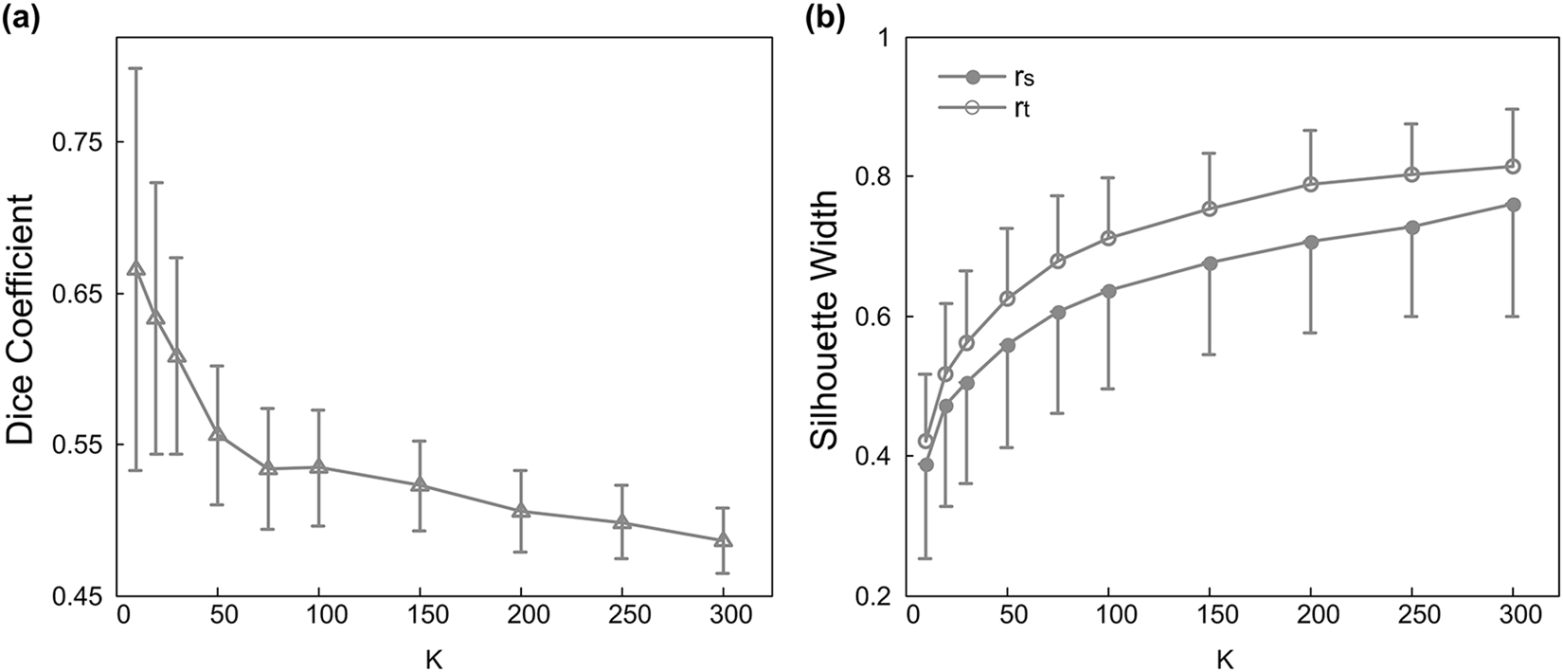
Comparison of atlases of different cluster numbers in: (**a**) reproducibility as measured by Dice’s coefficient, (**b**) functional homogeneity as measured by r_t_ and r_s_ silhouette widths. Symbols represent the mean and error bars indicate the standard deviation.

We first evaluated how cluster number influences the reproducibility of parcellation, as measured by Dice’s coefficient^46^. The value range of Dice’s coefficients in our results is comparable to previous parcellation study of the cerebral cortex using functional connectivity, supporting of our application in the cerebellum^37^ (Fig. 3a). In general, the Dice’s coefficient decreases when the number of clusters increases. This trend likely reflects increased spatial variability as clusters become smaller and sparser. This decrease in Dice’s coefficients slows down after cluster number reaches 100 (Fig. 3a).

We then evaluated the effect of cluster number on the functional homogeneity of clusters, as quantified by silhouette width. As expected, both r_t_ and r_s_ silhouette widths increase with the number of clusters (Fig. 3b), reflecting improved homogeneity with smaller cluster size. Again, this increasing trend shows down around cluster number of 100 and atlases with 100 or more clusters show good silhouette widths (Fig. 3).

### Comparison to existing cerebellar atlases

To further evaluate our functional atlases, we compared them against four existing cerebellar atlases: the Spatially Unbiased Atlas Template (28 parcels), Shen’s functional parcellation (46 parcels), Buckner’s 17 networks (17 parcels) and Buckner’s 34 networks (34 parcels) atlas, in terms of cluster homogeneity, accuracy of functional connectivity representation and individual identification. To avoid further potential biases introduced by different cluster numbers, we conducted these comparisons between existing atlases and our functional atlases with the same cluster numbers. Specifically, the statistical comparisons between our functional atlas and existing atlas were conducted by paired t-tests on each evaluation metric across all subjects. Bonferroni correction was applied in cases of multiple comparisons.

#### Cluster homogeneity

**Figure 4:**
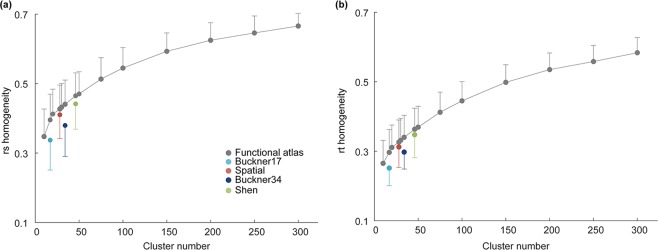
Comparison of cluster homogeneity between our atlases (gray symbol) and four existing cerebellar atlases (Buckner’s 17 networks atlas - light blue, Buckner’s 34 networks atlas - dark blue, Spatial: the Spatially Unbiased Atlas Template - red, Shen: Shen’s functional parcellation - green). Symbols represent the mean and error bars indicate the standard deviation across subjects.

For each atlas, the cluster homogeneity is measured by the average temporal (r_t_) and spatial (r_s_) correlation between pairs of voxels within each cluster; these values are then averaged across all clusters for each subject in the ‘validation’ group (Fig. 4). Our functional atlases show significantly higher temporal and spatial homogeneity than Buckner’s 17 networks (paired t-test, Bonferroni-corrected *P* < 5 × 10^−4^), Buckner’s 34 networks (paired t-test, Bonferroni-corrected *P* < 5 × 10^−4^), the Spatially Unbiased Atlas Template (paired t-test, Bonferroni-corrected *P* < 5 × 10^−3^) and Shen’s functional parcellation (paired t-test, Bonferroni-corrected *P* < 5 × 10^−3^), when the numbers of cluster are equal (Fig. 4). In addition, as both r_t_ and r_s_ homogeneity increase with the number of clusters, functional atlases using 100 or more clusters offer further improved cluster homogeneity.

#### Accuracy of functional connectivity representation

**Figure 5:**
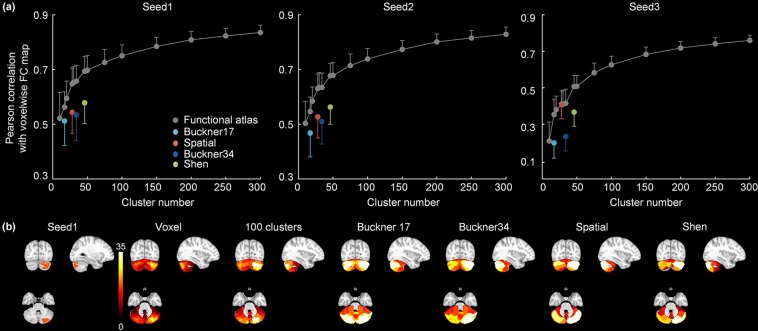
Comparison of (**a**) accuracy of functional connectivity representation for our atlases (gray symbol) and other four commonly-used atlases (Buckner’s 17 networks atlas - light blue, Buckner’s 34 networks atlas - dark blue, Spatial: the Spatially Unbiased Atlas Template - red, Shen: Shen’s functional parcellation - green). Symbols represent the mean and error bars indicate the standard deviation; (**b**) functional connectivity maps for seed1: based on voxel-wise data, our 100 cluster atlas, Buckner’s 17 networks atlas, Buckner’s 34 networks atlas, the Spatially Unbiased Atlas Template and Shen’s functional parcellation. Colors represent z-scores.

We next examined how well the atlases could be used to identify and represent functional connectivity networks. We selected three seeds within the cerebellum based on group ICA results: two seeds are located in Crus I/II and one in HVIII/HIX regions. The accuracy of functional connectivity representation is quantified by its similarity to the voxel-wise functional connectivity maps. As expected, as the number of clusters increases, functional connectivity maps based on our atlases are more accurate in representing the voxel-wise functional connectivity maps (Fig. 5a). Across all three seed-based networks, our functional atlases show significantly higher representation accuracy than the Buckner’s 17 networks (paired t-test, Bonferroni-corrected *P* < 0.05), Buckner’s 34 networks atlas (paired t-test, Bonferroni-corrected *P* < 5 × 10^−8^) and Shen’s functional parcellation (paired t-test, Bonferroni-corrected *P* < 5 × 10^−9^), when numbers of cluster are equal. Our 28-cluster atlas has significantly higher representation accuracy than the Spatially Unbiased Atlas Template for functional connectivity map based on seed1 and seed2 (paired t-test, Bonferroni-corrected *P* < 5 × 10^−8^) (Fig. 5a). The representation accuracy is further improved when using higher cluster numbers such as 100 (Fig. 5b).

#### Individual identification

**Figure 6:**
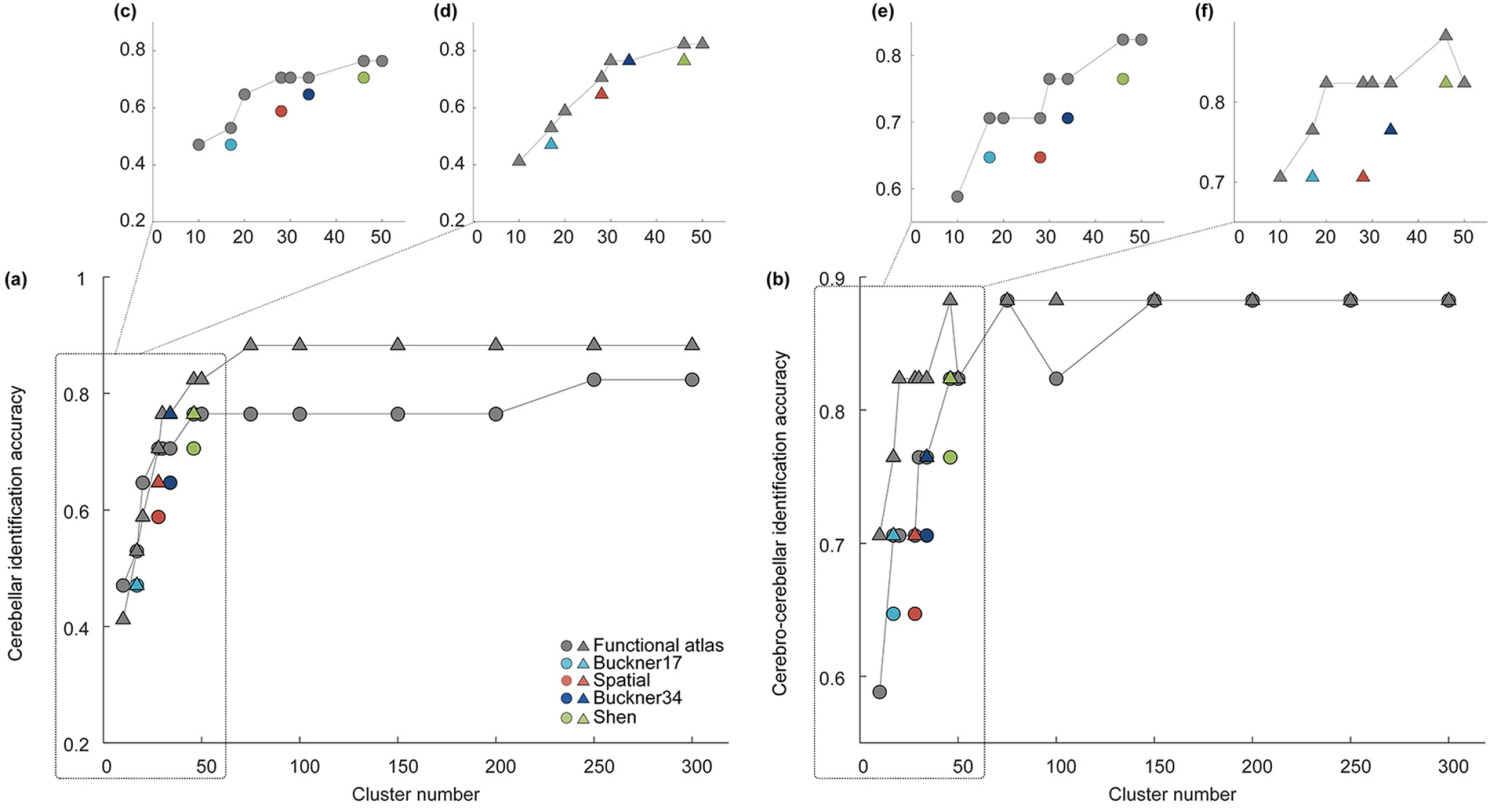
Comparison of identification accuracy across two sessions in ‘validation’ group for our (gray symbol) and four existing atlases (Buckner’s 17 networks atlas - light blue, Buckner’s 34 networks atlas - dark blue, Spatial: the Spatially Unbiased Atlas Template - red, Shen: Shen’s functional parcellation - green) using (**a**) cerebellar functional connectivity or (**b**) cerebro-cerebellar functional connectivity for individual identification analyses. Symbols (circle or triangle) indicate when the first session or second session was used as the target set (with the other group serving as the database set).

Functional connectivity of the cerebral cortex has been recently shown to be unique to individual and can be used for individual identification, using a fingerprint identification analysis^22^. We here examined whether functional connectivity of the cerebellum, derived with cerebellar ROI atlases, also manifest unique patterns between individuals. We conducted fingerprint identification analyses using either functional connectivity within the cerebellum alone (cerebellar functional connectivity, Fig. 6a) or functional connectivity between the cerebellar and cerebral cortex (cerebro-cerebellar functional connectivity, Fig. 6b).

Both types of functional connectivity yield high individual identification accuracy when cluster number is greater than 100 (Fig. 6). Identification results based on the cerebellar functional connectivity are poor at low cluster number (Fig. 6a), suggesting parcellation is insufficient at this resolution. Furthermore, for both cerebellar and cerebro-cerebellar functional connectivity-based identification analyses, our functional atlases mostly show higher accuracy than Shen’s functional parcellation, the Buckner’s networks and the Spatially Unbiased Atlas Template with the same cluster numbers (Fig. 6c–f). Notably, the improvement over the Spatially Unbiased Atlas Template is especially substantial (Table 1, bold), further supporting that morphology-based atlas might fall short at mapping function or connectivity of the cerebellum.

**Table 1:** Comparison of individual identification accuracy between existing atlases and our functional atlases with the same cluster number using either cerebellar connectivity or cerebro-cerebellar connectivity.

	Cerebellar		Cerebro-cerebellar	
	(Session 1)	(Session 2)	(Session 1)	(Session 2)
Buckner 17	0.4706	0.4706	0.6471	0.7059
Functional (17)	0.5294	0.5294	0.7059	0.7647
Improvement	5.88%	**5**.**88%**	5.88%	5.88%
Buckner 34	0.6471	0.7647	0.7059	0.7647
Functional (34)	0.7059	0.7647	0.7647	0.8235
Improvement	5.88%	0%	5.88%	5.88%
Shen	0.7059	0.8235	0.7647	0.8235
Functional (46)	0.7647	0.8235	0.8235	0.8824
Improvement	5.88%	0%	5.88%	5.88%
Spatial	0.5882	0.6471	0.6471	0.7059
Functional (28)	0.7059	0.7059	0.7059	0.8235
Improvement	**11**.**77%**	**5**.**88%**	5.88**%**	**11**.**77%**

The data group used for the target set is indicated in bracket. The most substantial improvement is highlighted in bold.

### Task fMRI analysis

**Figure 7:**
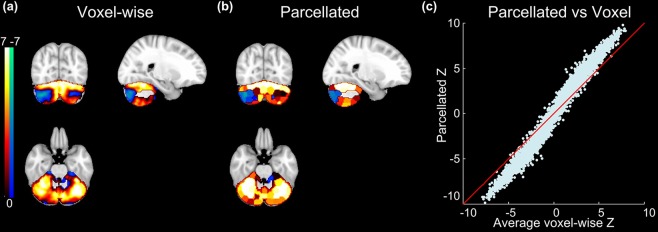
(**a**) Example voxel-wise and (**b**) parcellated z statistics maps derived from Gambling punish contrast. (**c**) The correlation between the averaged voxel-wise and parcellated z statistics (Points are 100 clusters × 86 contrasts).

Finally, we evaluated how well our cerebellar atlas could support statistical analyses of task fMRI activations. We performed this evaluation on the task fMRI datasets from HCP, which contain seven tasks (working memory, gambling, motor, language, social cognitive, relational processing and emotion processing) and 86 task contrasts (47 unique, 39 sign reversed)^33^. Comparing with voxel-wise analyses, functional activations based on our 100-cluster atlas reveal similar spatial patterns (Fig. 7a,b, Gambling punish contrast as an example), suggesting the average time series of each cluster can effectively represent all the voxels’ time series. We then correlated the average voxel-wise z statistics against the parcellated z statistics for all 86 task contrasts (100 clusters × 86 task z statistics) and found parcellated time series improved statistics over voxel-wise time series (Fig. 7c). Therefore, our results demonstrated that analyses based on parcellations, by averaging fMRI time series within the parcels, can improve the signal-to-noise ratio and increase z statistics for task fMRI studies, consistent with the report on parcellation of the cerebral cortex^1^. Thus, this task fMRI analysis further reveals that our functional atlas contains functionally homogenous clusters.

**Figure 8:**
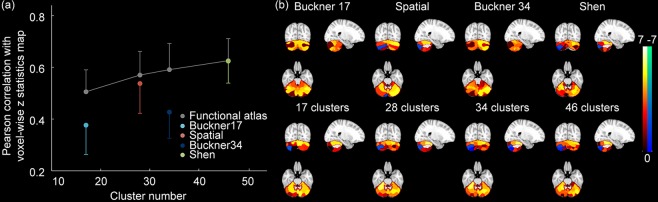
(**a**) Comparison of similarity of z statistics maps for our atlases (gray symbol) and other four commonly-used atlases (Buckner’s 17 networks atlas - light blue, Buckner’s 34 networks atlas - dark blue, Spatial: the Spatially Unbiased Atlas Template - red, Shen: Shen’s functional parcellation - green). Symbols represent the mean and error bars indicate the standard deviation. (**b**) Example atlas-based z statistics maps derived from Gambling punish contrast.

We next compared our functional atlas against four existing cerebellar atlases in terms of task fMRI analysis. The accuracy of z statistics maps is quantified by its similarity to the voxel-wise z statistics maps. Specifically, our functional atlases show significantly higher accuracy than the Buckner’s 17 networks (paired t-test, *P* < 5 × 10^−17^), Buckner’s 34 networks atlas (paired t-test, *P* < 5 × 10^−33^), and the Spatially Unbiased Atlas Template (paired t-test, *P* < 5 × 10^−5^), when numbers of cluster are equal (Fig. 8a). With 46 clusters, our atlas showed comparable performance with Shen’s functional parcellation (Fig. 8a). In addition, comparing with existing atlases, functional activations based on our functional atlases reveal relatively similar spatial patterns with voxel-wise z statistics maps (Figs 7a and 8b, Gambling punish contrast as an example), suggesting the superiority of our cerebellar parcellation in detecting functional activations for task fMRI.

## Discussion

Neuroscientists are increasingly intrigued by the functional complexity and diversity of the cerebellum. Traditionally thought as a structure dedicated to motor function, the cerebellum is now known to be activated by a variety of non-motor tasks. To probe the diverse non-motor functions of cerebellum, it is essential to identify its functional subdivisions. While previous cerebellar atlases mostly parcellated the cerebellum based on its exceptionally regular morphology, assuming a direct mapping between morphology and function^18,19^, we here suggest that a connectivity-based parcellation could substantially improve functional representation of the cerebellum. Building upon the earlier works on functional connectivity networks in the cerebellum^16^, this atlas provides a more detailed parcellation of the cerebellar subdivisions that are spatially and functionally homogenous. Quantitative comparisons demonstrated our atlas is superior to existing cerebellar atlases in cluster homogeneity, accuracy of functional connectivity representation and individual identification. In addition, the value ranges of Dice coefficient, silhouette width, and other metrics are comparable to previous parcellation study of the cerebral cortex using functional connectivity, further supporting our application in the cerebellum^37^.

In our study, we employed a two-step clustering method to generate cerebellar parcellation, including generating subject specific similarity matrices and finalizing the clustering at the group level^37^. In our study, we generated the subject specific similarity matrices using r_t_ metric to ensure the temporal homogeneity within a cluster. While r_s_ metric was not used in the initial clustering, it still shows similar performance as r_t_ in assessing cluster homogeneity (Figs 3b and 4), suggesting that atlases generated via r_t_ similarity metric could support both temporal and spatial homogeneity^37^. We found that r_s_ silhouette width has larger variability across subjects (Fig. 3b), consistent with previous study^37^. Furthermore, there are two available methods for group level clustering - group mean and two-level clustering methods. Although the two-level clustering might perform slightly better than the group mean clustering, it requires a greater computational expense^37^. As our study used a relatively large datasets of 114 resting state fMRI datasets each with 1200 volumes, we chose the group mean clustering method to improve computational efficiency.

A key goal of parcellation scheme is to reduce the total number of parcels to improve the computational efficiency over voxel-wise calculation. However, as the cluster number decreases, the clusters become larger, and unavoidably less representative of the individual voxels. Therefore, a suitable parcellation solution has to balance between computational efficiency and spatial resolution. In our results, atlases with 100 or less clusters have relatively high Dice coefficients but also high standard deviation compared to atlases with more clusters (Fig. 3a). On the other hand, both r_t_ and r_s_ silhouette width metrics are poor for atlases with less clusters and increase with cluster number (Fig. 3b). Furthermore, results of cluster homogeneity, accuracy of functional connectivity representation and individual identification based on our atlases are poor at cluster number lower than 100 (Figs 4–6), suggesting insufficient parcellation at this resolution. Overall, our study provides a multi-resolution set of atlases with robust performance in functional connectivity-based analyses (100–300). However, we suggest that researchers should choose the atlas resolution to use according to the specific research questions: high cluster number could be more useful when fine scale representation and homogeneity is desired, and lower cluster number could be used when computational efficiency is desired without substantial loss of information. Nonetheless, most existing parcellations with less than 50 regions might be suboptimal for representing the functional diversity of the cerebellum.

The understanding of the cerebellum function has been considerably advanced by functional neuroimaging studies of language processing, working memory, executive function and emotion processing functions^6,8,9,11,13,15,21,51^. Interestingly, these studies often revealed functional activations covering a subportion of a lobule or sometimes across two lobules. In addition, the cerebellar components of functional connectivity networks do not appear to follow the morphological boundaries of the cerebellum^16^. This disconnection between function and morphology, however, has not been well recognized and addressed. Functional neuroimaging studies still reply on the gross anatomical nomenclature to define activations in the cerebellum and recent efforts to improve cerebellar atlas mostly focus on automated identification of the anatomical boundaries of cerebellar lobules^18,19^. Furthermore, our previous clinical study revealed that cerebellar subregions targeted by Alzheimer’s disease and behavioural variant frontotemporal dementia (bvFTD) are all within subportion of lobule, suggesting the necessity of functional parcellation of cerebellum of clinical study^52^. Thus, our study provided solid evidence that anatomical atlas does not well represent the function and connectivity of the cerebellum, and support the connectivity-based parcellation in future work (Figs 4–6). In addition, consistent with functional neuroimaging studies of cerebellum function, our functional parcellation does not fully match the morphology of cerebellum. For example, clusters in our 100-cluster atlas often cover across two lobules, while clusters in our 300-cluster atlas often cover a subportion of a lobule, further suggesting the disconnection between the function and morphology of cerebellum.

In addition to anatomical atlas of cerebellum, our functional parcellation also shows superiority to existing functional parcellations in regards to cluster homogeneity and functional connectivity-based analyses. Our data-driven method cooperates both spatial constraint and functional connectivity information together to generate functional cerebellar atlas. While the spatial constraint appears to dictate much of the size of the ROIs, functional information refines the boundaries of ROIs. Note that a key point is that our clusters are spatially coherent and thus are different from those spatial distributed local network nodes or large-scale networks, such as Buckner’s network^16^. However, Buckner’s study used cerebellar-cortical functional connectivity as metric to investigate the organization of cerebrocerebellar circuits and delineate the functional boundaries of the cerebellar-cortical network, resulting in gross networks in the parcellation and worse performance than our atlas in regards to cluster homogeneity and functional connectivity-based analyses. On the other hand, compared to Shen’s whole brain functional parcellation^23^, our method only focuses on whole cerebellar signals, where the similarity matrix is calculated within cerebellum, thus resulting in more functionally homogenous clusters within cerebellum. However, as Shen’s work aimed to identify the whole-brain parcellation, their weight matrix was calculated among the whole-brain. Thus, the cerebellar clusters in their parcellation were determined and affected by the connections between cerebellum and cerebral cortex, probably resulting in the superiority of our cerebellar parcellation than this subpart of Shen’s whole brain parcellation in the comparison.

Several caveats of our results should be noted. First, the individual identification using cerebro-cerebellar functional connectivity outperforms that using cerebellar connectivity, suggesting that cerebro-cerebellar functional connectivity could provide additional information about the function of individual brains. While our atlas is still superior in the specific goal of this current study, to characterize functionally homogeneous and spatially coherent clusters in the cerebellum, rather than to delineate the cerebellar-cortical network as in the Buckner study^16^, future work could further extend to incorporate cerebro-cerebellar connectivity into the parcellation. Second, our preprocessing pipeline includes several steps, which could introduce spatial smoothing, including motion correction, normalization to standard space and spatial smoothing filter. While these steps should improve the correspondence of cerebellum regions across subjects, smoothing could induce correlation and impact the clustering results. Nonetheless, to minimize the impact of smoothing for the cerebellum, a region with high neural density, we used only 2 mm FWHM Gaussian kernel in the spatial smoothing step. Future work could investigate the impact of preprocessing on clustering results. Third, recent work identified functional whole-brain parcellation constrained by AAL boundaries^53^, where each resulting cluster can be linked with an anatomical annotation from the AAL template. In the cerebellum, however, there is disconnection between function and morphology of cerebellum, as discussed above. Thus, we applied our functional parcellation without the constraint and potential bias by the anatomical boundaries. Further work is needed to determine the relationship between functional and anatomical divisions. Fourth, recent neuroimaging studies have demonstrated age and gender effects on brain connectivity. Thus, age and gender would potentially affect the functional connectivity in the cerebellum and functional connectivity-based parcellation^54,55^, which is a promising direction to investigate in the future.

In conclusion, the data-driven method based on normalized cut spectral clustering (NCUT) algorithm successfully parcellated cerebellum into spatially and functionally homogeneous clusters using resting state functional connectivity. While previous studies have defined functional connectivity-based cerebellar network or whole brain parcellation including cerebellum coverage^16,22,23^, our parcellation reveal superior performance in cluster homogeneity, accuracy of functional connectivity representation and individual identification analyses. Hence our functional parcellation provides a valuable tool for dimensionality reduction in functional connectivity and activation analyses in basic and clinical research. While we controlled for potential biases introduced by cluster number, the optimal cluster number for specific analysis depends on the specific research questions. In addition, the current parcellation only incorporated resting state functional connectivity measures, and it could be further refined by including multimodal images of cerebellum in the future.

## Data Availability

The resting state and task fMRI datasets are available from HCP (https://www.humanconnectome.org/study/hcp-young-adult). The functional atlases with different cluster numbers are available on our website (www.neuroguo.com/resources/).

## Supplementary information

Supplementary information
