# Supplementary material for: A connectivity-based parcellation improved functional representation of the human cerebellum
Source: Sci Rep. 2019 Jun 24;9:9115. doi: 10.1038/s41598-019-45670-6 (PMC6591283; doi:10.1038/s41598-019-45670-6)
Supplement: Supplementary file 1 — Supplementary information [file 41598_2019_45670_MOESM1_ESM.pdf]

A connectivity-based parcellation improved functional representation of the human cerebellum

Yudan Ren[1,2], Lei Guo[1], Christine Cong Guo[2*]

[1]School of Automation, Northwestern Polytechnical University, Xi'an, China;

[2]QIMR Berghofer Medical Research Institute, Brisbane, Australia.

[*]corresponding authors.

Results

1. Comparison to existing cerebellar atlases using 'evaluation' group

1.1 Cluster homogeneity

Our functional atlases show significantly higher temporal and spatial homogeneity than Buckner's 17 networks (paired t-test, Bonferroni-corrected $P<5\times10^{-27}$), Buckner's 34 networks (paired t-test, Bonferroni-corrected $P<5\times10^{-10}$), the Spatially Unbiased Atlas Template (paired t-test, Bonferroni-corrected $P<1\times10^{-16}$) and Shen's functional parcellation (paired t-test, Bonferroni-corrected $P<5\times10^{-7}$), when the numbers of cluster are equal (Supplementary Fig. 1). In addition, consistent with main results, as both r_t and r_s homogeneity increase with the number of clusters, functional atlases using 100 or more clusters offer further improved cluster homogeneity.

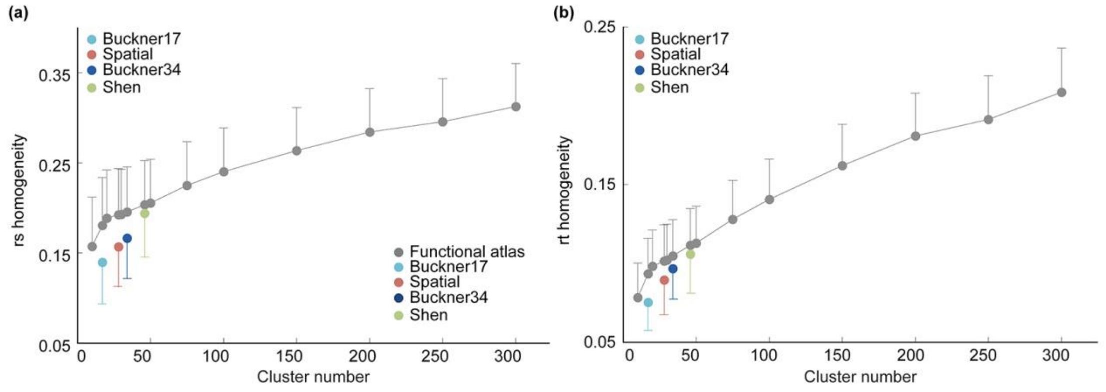

Supplementary Fig. 1 Comparison of cluster homogeneity between our atlases (gray symbol) and four existing cerebellar atlases (Buckner's 17 networks atlas - light blue, Buckner's 34 networks atlas - dark blue, Spatial: the Spatially Unbiased Atlas Template - red, Shen: Shen's functional parcellation - green) in

‘evaluation’ group. Symbols represent the mean and error bars indicate the standard deviation across subjects.

1.2 Accuracy of functional connectivity representation

We selected the same three seeds within the cerebellum as main results. Consistent with main results, as the number of clusters increases, functional connectivity maps based on our atlases are more accurate in representing the voxel-wise functional connectivity maps (Supplementary Fig. 2a). Across all three seed-based networks, our functional atlases show significantly higher representation accuracy than the Spatially Unbiased Atlas Template (paired t-test, Bonferroni-corrected $P<5\times10^{-4}$), Buckner's 34 networks atlas (paired t-test, Bonferroni-corrected $P<5\times10^{-12}$) and Shen's functional parcellation (paired t-test, Bonferroni-corrected $P<1\times10^{-31}$), when numbers of cluster are equal. Our 17-cluster atlas has significantly higher representation accuracy than the Buckner's 17 networks for functional connectivity map based on seed2 (paired t-test, Bonferroni-corrected $P<5\times10^{-9}$) (Supplementary Fig. 2a). The representation accuracy is further improved when using higher cluster numbers such as 100 (Supplementary Fig. 2b).

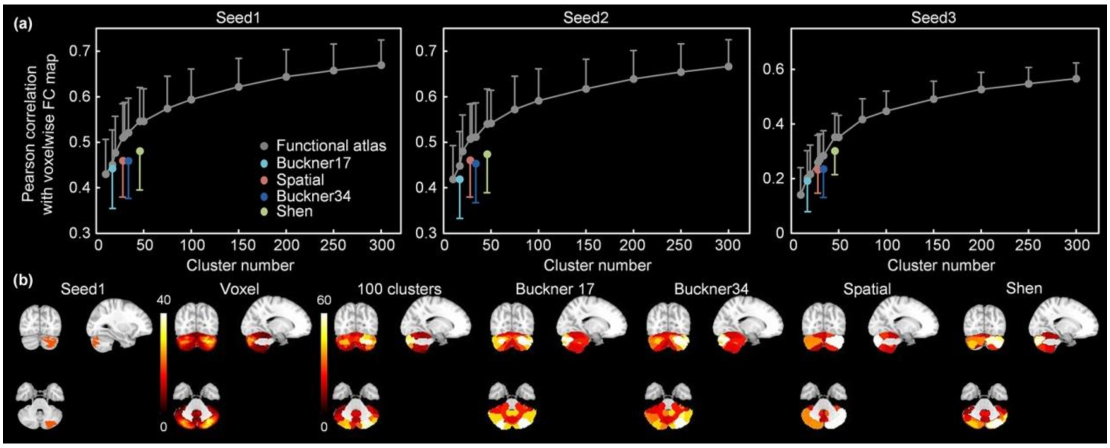

Supplementary Fig. 2 Comparison of (a) accuracy of functional connectivity representation for our atlases (gray symbol) and other four commonly-used atlases (Buckner's 17 networks atlas - light blue, Buckner's 34 networks atlas - dark blue, Spatial: the Spatially Unbiased Atlas Template - red, Shen: Shen's functional parcellation - green) in 'evaluation' group. Symbols represent the mean and error bars indicate the standard deviation; (b) functional connectivity maps for seed1: based on voxel-wise data, our 100 cluster atlas, Buckner's 17 networks atlas, Buckner's 34 networks atlas, the Spatially Unbiased Atlas Template and Shen's functional parcellation. Colors represent z-scores.

1.3 Individual identification

We conducted fingerprint identification analyses between 'parcellation' group and 'evaluation' group using either functional connectivity within the cerebellum alone (cerebellar functional connectivity, Supplementary Fig. 3a) or functional connectivity between the cerebellar and cerebral cortex (cerebro-cerebellar functional connectivity, Supplementary Fig. 3a).

Both types of functional connectivity yield high individual identification accuracy when cluster number is greater than 100 (Supplementary Fig. 3a). Identification results based on the cerebellar functional connectivity are poor at low cluster number (Supplementary Fig. 3a), suggesting parcellation is insufficient at this resolution. Furthermore, for both cerebellar and cerebro-cerebellar functional connectivity-based identification analyses, our functional atlases show higher accuracy than Shen's functional parcellation, the Buckner's networks and the Spatially Unbiased Atlas Template with the same cluster numbers (Supplementary Fig. 3c-f). Notably, the improvement over the Spatially Unbiased Atlas Template is especially substantial (Supplementary Table. 1, bold), further supporting that morphology-based atlas might fall short at mapping function or connectivity of the cerebellum.

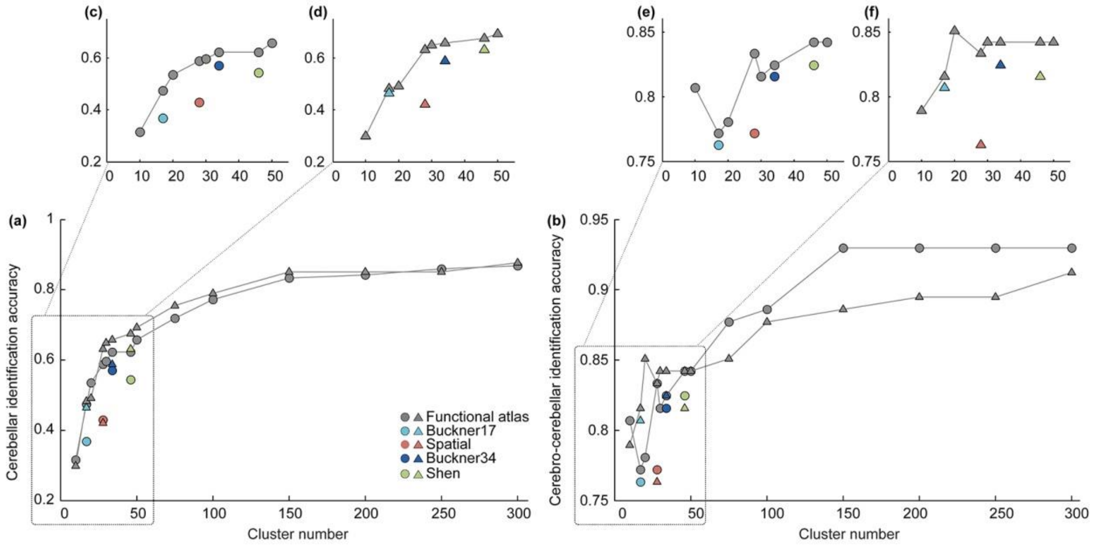

Supplementary Fig. 3 Comparison of identification accuracy across two sessions ('parcellation group' and 'evaluation' group) for our (gray symbol) and

four existing atlases (Buckner's 17 networks atlas - light blue, Buckner's 34 networks atlas - dark blue, Spatial: the Spatially Unbiased Atlas Template - red, Shen: Shen's functional parcellation - green) using (a) cerebellar functional connectivity or (b) cerebro-cerebellar functional connectivity for individual identification analyses. Symbols (circle or triangle) indicate when the 'parcellation group' or 'evaluation' group was used as the target set (with the other group serving as the database set).

Supplementary Table. 1 Comparison of individual identification accuracy between existing atlases and our functional atlases with the same cluster number using either cerebellar connectivity or cerebro-cerebellar connectivity between 'parcellation' group and 'evaluation' group. The data group used for the target set is indicated in bracket. The most substantial improvement is found over the Spatial atlas as highlighted in bold.

	Cerebellar		Cerebro-cerebellar	
	(parcellation)	(evaluation)	(parcellation)	(evaluation)
Buckner 17	0.3684	0.4649	0.7632	0.8070
Functional (17)	0.4737	0.4825	0.7719	0.8158
Improvement	10.53%	1.76%	0.87%	0.88%
Buckner 34	0.5702	0.5877	0.8158	0.8246
Functional (34)	0.6228	0.6579	0.8246	0.8421
Improvement	5.26%	7.02%	0.88%	1.75%

Shen	0.5439	0.6316	0.8246	0.8158
Functional (46)	0.6228	0.6754	0.8421	0.8421
Improvement	7.89%	4.38%	1.75%	2.63%
Spatial	0.4298	0.4211	0.7719	0.7632
Functional (28)	0.5877	0.6316	0.8333	0.8333
Improvement	**15.79%**	**21.05%**	**6.14%**	**7.01%**

Supplementary Table. 2 IDs of all the subjects in HCP resting state fMRI data.

Subject ID							
100408	102816	205826	190031	130922	151526	545345	541943
101915	105216	211720	196750	140824	154431	705341	570243
106016	111009	239944	198451	148032	159340	904044	586460
111514	120111	245333	224022	148335	162733	978578	680957
111716	126325	280739	246133	160123	163129	765056	704238
122317	130316	371843	390645	178950	176542	871964	751348
127933	133625	429040	497865	199655	188347	899885	761957
959574							
